# Atypical Endometrial Hyperplasia and Unexpected Cancers at Final Histology: A Study on Endometrial Sampling Methods and Risk Factors

**DOI:** 10.3390/diagnostics10070474

**Published:** 2020-07-13

**Authors:** Luca Giannella, Giovanni Delli Carpini, Francesco Sopracordevole, Maria Papiccio, Matteo Serri, Giorgio Giorda, Dimitrios Tsiroglou, Anna Del Fabro, Andrea Ciavattini

**Affiliations:** 1 Woman’s Health Sciences Department, Gynecologic Section, Polytechnic University of Marche, 60121 Ancona, Italy; luca.giannella@ospedaliriuniti.marche.it (L.G.); Giovanni.DelliCarpini@ospedaliriuniti.marche.it (G.D.C.); maria.papiccio@gmail.com (M.P.); serri.mat@gmail.com (M.S.); dimitrios.tsiroglou@ospedaliriuniti.marche.it (D.T.); 2 Gynecological Oncology Unit, Centro di Riferimento Oncologico-National Cancer Institute, 33081 Aviano, Italy; fsopracordevole@cro.it (F.S.); ggiorda@cro.it (G.G); anna.delfabro@cro.it (A.D.F.)

**Keywords:** atypical endometrial hyperplasia, endometrial cancer, risk factors, dilation and curettage, hysteroscopically guided biopsy, hysteroscopic endometrial resection

## Abstract

Background: Up to 40% of women with atypical endometrial hyperplasia (AEH) can reveal endometrial cancer (EC) at hysterectomy. The pre-operative endometrial sampling method (ESM) and some independent cancer predictors may affect this outcome. The present study aimed to compare the rate of EC at hysterectomy in women with AEH undergoing dilation and curettage (D&C), hysteroscopically-guided biopsy (HSC-bio), or hysteroscopic endometrial resection (HSC-res). The secondary outcome was to compare the reliability of ESMs in women showing independent variables associated with EC. Methods: Two-hundred-and-eight consecutive women with AEH and undergoing hysterectomy between January 2000 and December 2017 were analyzed retrospectively. Based on pre- and post-test probability analysis for EC, three ESMs were compared: D&C, HSC-bio, and HSC-res. Univariate and multivariate analyses were performed to assess risk factors predicting cancer on final histology. Finally, the patient’s characteristics were compared between the three ESM groups. Results: D&C and HSC-bio included 75 women in each group, while HSC-res included 58 women. Forty-nine women (23.6%) revealed cancer at hysterectomy (pre-test probability). Post-test probability analysis showed that HSC-res had the lowest percentage of EC underestimation: HSC-res = 11.6%; HSC-bio = 19.5%; D&C = 35.3%. Patient characteristics showed no significant differences between the three ESMs. Multivariate analysis showed that body mass index ≥40 (Odds Ratio (OR) = 19.75; Confidence Intervals (CI) 2.193–177.829), and age (criterion > 60 years) (OR = 1.055, CI 1.002–1.111) associated significantly with EC. In women with one or both risk factors, post-test probability analysis showed that HSC-res was the only method with a lower EC rate at hysterectomy compared to a pre-test probability of 44.2%: HSC-res = 19.96%; HSC-bio = 53.81%; D&C = 63.12%. Conclusions: HSC-res provided the lowest rate of EC underestimation in AEH, also in women showing EC predictors. These data may be considered for better diagnostic and therapeutic planning of AEH.

## 1. Background

The last classification of endometrial hyperplasia is the WHO 2014 Classification System, which defined only two categories of endometrial hyperplasia: non-atypical endometrial hyperplasia (benign hyperplasia) and atypical endometrial hyperplasia (AEH) or Endometrial Intraepithelial Neoplasia (EIN/well-differentiated carcinoma) [1]. The new 2014 WHO classification improves diagnosis reproducibility and clearly distinguishes between two different pathological entities (with and without atypia) managed differently because of the risk of progression [2].

AEH is a premalignant condition of not easy clinical management. Almost one in two women may already have concurrent endometrial cancer (EC), as well as up to 30% of cases can progress to cancer within 12 months [3,4,5]. Given these numbers, undertreatment is not uncommon [6]. Usually, these women do not always follow an oncological work-up, and definitive histology may reveal a disease stage that would have required more radical surgery [6]. However, it should be considered that conservative medical treatment may be an option after appropriate counseling in women of childbearing age [7]. Therefore, it is clear that an accurate diagnosis is pivotal for women candidates for surgery or conservative treatment.

The rate of unexpected cancer in AEH may be affected by some EC predictors, and the endometrial sampling method (ESM) used preoperatively [7,8,9,10,11,12]. Previous studies have shown that some cancer predictors in women with AEH are associated with concurrent EC [13,14]. Less is present in the literature about the diagnostic reliability of ESMs in women with AEH. Dilation and curettage (D&C) was the most used and studied ESM. Previous authors showed that ~30% of women with AEH at D&C revealed EC on hysterectomy [8]. 

Therefore, to date, it is unclear what is the most reliable ESM in women with AEH. Furthermore, it is not known if the presence of EC predictors can affect the reliability of ESMs.

The present study aimed to compare the rate of EC at hysterectomy in women with AEH undergoing D&C, or hysteroscopically-guided biopsy (HSC-bio), or hysteroscopic endometrial resection (HSC-res). The secondary outcome was to compare the reliability of ESMs in women showing independent variables associated with EC.

## 2. Methods

This retrospective observational study included women with a preoperative diagnosis of AEH at the Oncology Reference Center of Aviano and the University Hospital of Ancona, Italy, between January 2000 and December 2017. As this study was merely observational and involved data analysis from routine measurements, with no additional or experimental interventions, an institutional review board approval was not required. All patients provided written, informed consent to use their data for research purposes before any diagnostic or therapeutic procedure.

Data collection included women with AEH diagnosis and then undergoing major surgery. Our final histological reference standard was represented by hysterectomy. All included women had to have performed the surgery within six weeks of AEH diagnosis. The preoperative ESMs included D&C, HSC-bio, and HSC-res. All diagnostic hysteroscopies were performed without anesthesia in an outpatient setting and vaginoscopy with a saline solution as a distension medium and using a 5 mm continuous-flow sheath with a viewing angle of 30°. D&C and HSC-res were performed under general anesthesia. HSC-res used a 10 mm continuous-flow sheath with a viewing angle of 0°. D&C was performed in case of heavy vaginal bleeding, or insufficient endometrial sampling to HSC-bio that showed no focal endocavitary lesions. HSC-res was performed in case of HSC-bio failure with suspected focal endouterine lesion, or inadequate endometrial sampling to HSC-bio that showed a focal endocavitary lesion [15]. All procedures were performed by senior gynecologists with hysteroscopic skills. 

All histological samples were evaluated by dedicated pathologists who dealt with gynecological pathology. Our histological classification of AEH refers to the WHO 2014 Classification [1]. Histological samples performed before the introduction of the WHO 2014 Classification were revised accordingly to new categories. Women with previous events of AEH managed with medical treatment, or AEH diagnosis on endometrial polyps were excluded.

All data were collected from medical records. Patient characteristics taken into account were age (years), age at menarche (years), parity, body mass index (BMI = weight (kg)/height^2^ (m^2^)), presence of hypertension or diabetes, hereditary cancer syndromes, current hormonal therapy, smoking habit, tamoxifen users, and presence of abnormal uterine bleeding.

Three endometrial sampling methods were compared: D&C, HSC-bio, and HSC-res. Based on final histology at hysterectomy, the percentage of EC underestimation was assessed between the study groups. In this regard, post-test probability analysis was performed using the likelihood ratio. Patient characteristics were compared between the three ESM groups. Then, women were divided into two groups: patients with or without cancer on final histology. Independent variables were assessed by univariate and multivariate analysis. Endometrial carcinoma staging has been reported. Women were classified as either low risk (stage 1: grading 1 or 2 and myometrial invasion <50%), intermediate risk (stage 1: myometrial invasion >50% or grading 3 and myometrial invasion <50%), or high risk (stage 1: grading 3 and myometrial invasion >50%, or stage 2+) [16]. Finally, the type of surgery was reported: surgical approach (laparotomic, laparoscopic, or vaginal), with or without salpingo-oophorectomy.

The Kolmogorov–Smirnov test was used as the test for normal distribution. Continuous variables were expressed as median and interquartile range. Qualitative variables were expressed as numbers and percentages. Univariate logistic regression analysis was used to test all studied independent variables. The nonparametric Mann–Whitney U-test for two independent samples was used for not normally distributed values. Comparisons between categorical variables were performed using the Chi-squared test. Non-normally distributed continuous variables between 3 or more groups were compared using the Kruskal–Wallis test. Multivariate logistic regression analysis was used to identify variables that associated significantly with EC. We included explanatory variables that showed a *p*-value ≤ 0.25 in the univariate model [17]. Receiver operating characteristic (ROC) curve analysis was used to calculate the best cut-off value (criterion) for continuous variables associated with cancer on final histology. After considering our disease prevalence (all cases of EC) as the pretest probability for malignant endometrial pathology, the likelihood ratio was used to calculate the post-test odds from the pretest odds of disease: post-test odds = pretest odds x likelihood ratio. The relation between odds and probability is as follows: odds = P/(1 − P) and P = odds/(1 + odds). Using these equations, we could calculate the post-test probability of disease from the pretest probability of disease [18,19]. 

All statistical analyses were performed using MedCalc Statistical Software Version 19.0.3 (MedCalc Software bvba, Ostend, Belgium; https://www.medcalc.org; 2019). *p* < 0.05 was considered to indicate statistical significance.

## 3. Results

Two-hundred-and-eight consecutive women with pre-operative AEH and undergoing hysterectomy were included in this study over eighteen years. 

Histological examination revealed the presence of 49 (23.6%) women with endometrial cancer at hysterectomy. One-hundred-and-fifty women (72.1%) underwent a total laparohysterectomy with bilateral salpingo-oophorectomy; 14 women (6.7%) experienced a total laparoscopic hysterectomy with bilateral salpingo-oophorectomy; seven women (3.4%) underwent a total vaginal hysterectomy with bilateral salpingo-oophorectomy; 23 women (11.1) underwent a total laparohysterectomy; nine women (4.3%) experienced a total laparoscopic hysterectomy; finally, five women (2.4%) underwent a total vaginal hysterectomy. Women with EC were classified as follows, 37 low risk (75.5%), 10 intermediate risk (20.4%), two high risk (4%) (one woman with stage 1 EC, and one woman with stage 3a EC). Women with EC underwent 39 (79.6%) total laparohysterectomies with bilateral salpingo-oophorectomy, four (8.2%) total laparoscopic hysterectomies with bilateral salpingo-oophorectomy, one (2%) total vaginal hysterectomy with bilateral salpingo-oophorectomy, one (2%) total laparohysterectomy without salpingo-oophorectomy, and four (8.2%) total laparoscopic hysterectomies without salpingo-oophorectomy. 

Patient characteristics showed no significant differences between the three ESM groups (Table 1). Concerning ESMs, 75 women performed D&C, 75 women performed HSC-bio, and 58 women performed HSC-res. The lowest percentage of EC at hysterectomy occurred in women undergoing HSC-res (14.3%) (*p* = 0.0037) (Table 2). More precisely, from a pre-test probability of 23.6% for EC, post-test probability analysis showed that HSC-res had the lowest percentage of EC underestimation: HSC-res = 11.6%; HSC-bio = 19.5%; D&C = 35.3% (Table 3).

Patient characteristics showed no significant differences between women with or without cancer at final histology about parity, smoking habit, comorbidity, hereditary cancer syndromes, history of previous breast cancer, tamoxifen users, hormonal therapy use, age at menarche, presence of abnormal uterine bleeding (Table 4). Conversely, significant differences were present about age (*p* = 0.010), menopausal status (*p* = 0.028), and BMI (*p* = 0.025) (Table 4). Multivariate analysis showed that age (criterion > 60 years) (adjusted odds ratio = 1.055; 95% confidence intervals 1.002–1.111) and BMI ≥ 40 (adjusted odds ratio = 19.751; 95% confidence intervals 2.193–177.829) associated significantly with EC at hysterectomy (Table 5). 

Finally, women with one or both risk factors (BMI ≥ 40 and/or age > 60 years) showed a percentage of EC at hysterectomy of 44.2% (Table 6). In these women, post-test probability analysis showed the following percentage of EC underestimation: HSC-res = 19.96%; HSC-bio = 53.81%; D&C = 63.12 (Table 6).

## 4. Discussion

The present study showed that HSC-res provided the lowest rate of EC underestimation in AEH, also in women showing EC predictors. 

Our percentage of unexpected cancers at final histology was 23.6%. This result is in line with what is reported in the literature, with percentages ranging from 20% to 40% (3, 7, 13, 14). A recent meta-analysis showed an EC rate of 31% in women with a pre-operative diagnosis of AEH (12). In our case, the higher percentage of women undergoing HSC-res increased the diagnostic accuracy of AEH by decreasing EC instances. Recently, Erdem et al. also showed an EC rate of 25.1% in 227 women with AEH confirming our results [20].

Pre-operative AEH diagnosis is a challenging clinical condition because up to 40% of women may already have concurrent endometrial cancer [7]. The risk of undertreatment is not negligible, and the surgical choice may not be appropriate, as well as the diagnostic work-up. In the EC case, further diagnostic steps may be needed for better clinical staging (i.e., magnetic resonance imaging) [16]. The surgery of an AEH involves a hysterectomy, while the gold standard for endometrial cancer is total hysterectomy with bilateral salpingo-oophorectomy (strength of recommendation: A) [16]. In intermediate risk women, a possible lymphadenectomy should be discussed (strength of recommendation: C), while in high-risk patients, lymph node dissection is recommended (strength of recommendation: B) [16]. Sentinel lymph node dissection could be feasible (strength of recommendation: D) [16]. Although choosing an intraoperative frozen section can help, its poor reliability has been demonstrated [21]. Therefore, the risk of mismanagement can be high for women with AEH. In the present study, ~25% of women with EC were classified as intermediate- or high-risk patients, and more than 10% of women with EC underwent a hysterectomy without salpingo-oophorectomy. These data also show the importance of an accurate preoperative diagnosis for those women candidates for conservative treatment. In this case, the false negatives could have a more significant impact.

With this background, several studies have evaluated possible independent variables associated with occult endometrial cancer in women with AEH. Matsuo et al. showed that “older age, obesity, diabetes mellitus, and complex atypical hyperplasia are predictive of concurrent endometrial carcinoma in endometrial hyperplasia patients” [14]. A recent study showed that “endometrial stripe thickness and age were the strongest predictors of concurrent endometrial cancer at the time of hysterectomy for endometrial intraepithelial neoplasia” [13]. In line with these results, the present study showed an association between elevated BMI, older age, and EC at hysterectomy in women with AEH. 

There is a further and less studied factor affecting the rate of unexpected cancer in women with AEH: the preoperative ESM. The reliability of ESMs in AEH diagnosis is a current topic with no conclusive evidence. The studies in the literature have often compared outpatient techniques having an inappropriate histological reference standard, such as D&C. A recent very interesting meta-analysis evaluated for the first time three ESMs (D&C, HSC-bio, HSC-res) to assess the most accurate method in women with AEH [12]. It showed that HSC-res was the method with the lowest EC underestimation at hysterectomy: HSC-res = 5.8%, D&C = 32.7%, HSC-bio = 45.3%. Although their sample was extensive, including over 1000 women, HSC-res cases included only 23 women requiring further studies to confirm these findings [12]. The present study confirmed these results showing HSC-res as the most reliable ESM on a greater sample of AEH diagnosed by HSC-res. As previously explained, HSC-res is very likely to sample much more tissue under vision, resulting in a more reliable sampling method [12]. 

Our results showed D&C as the least accurate method. In the literature, there are studies showing the superiority of HSC-bio over D&C and vice versa in this area [22,23,24]. We want to emphasize that several authors have reported that in 60% of uterine curettages, less than half of the uterine cavity is sampled [25]. 

For the first time, we also analyzed the reliability of ESMs in women with one or both risk factors for EC. Moreover, in this case, HSC-res proved to be the most reliable ESM with a post-test probability of 19.9% from a pre-test probability of 44.2%. 

Although HSC-res was the most accurate method of endometrial sampling, some open questions in this area should be discussed. Compared to other methods, HSC-res has a longer learning curve, higher surgical risks, and higher costs. A recent meta-analysis, including 1015 patients undergoing diagnostic hysteroscopy, showed greater dissemination of endometrial cells in the peritoneal cavity of women with EC [26]. However, the prognostic value of positive peritoneal cytology is unclear. Ben-Arie et al. showed no significant differences in survival or recurrence rates in women with EC who underwent endometrial biopsy, uterine curettage, or hysteroscopy [27]. Therefore, if HSC-res is confirmed to be the most accurate method of endometrial sampling, a hypothetical diagnostic work-up should evaluate this risk–benefit ratio carefully.

Although our results may be of interest to the clinical practice, some considerations must be stressed. The most obvious conclusion could be that women with AEH should be directed to HSC-res to minimize occult cancer risk on hysterectomy, especially when cancer predictors are present. However, the recommended treatment for atypical endometrial hyperplasia is to perform a total hysterectomy (with bilateral salpingo-oophorectomy when possible) in women who do not wish to become pregnant. Considering a further sampling method in a patient candidate for surgery would only serve to delay definitive management. On the other hand, conservative treatment can be offered in women wishing to become pregnant, or non-candidates for surgery due to the presence of severe clinical contraindications. In this case, the use of ESM with the lowest underestimation of cancer can have its rationale. In such a scenario, D&C and HSC-bio could require an additional diagnostic step represented by HSC-res. Using a more reliable method could be more reassuring about conservative management’s choice. Based on our results, if conservative treatment is to be decided in women with EC predictors, HSC-res should be considered. Although this option does not rule out the possibility of having cancer, it significantly decreases the rate of false negatives. 

The present study has several limitations. A limitation is its retrospective nature. Some pre-surgical variables of importance, such as endometrial ultrasound evaluation, were not included. Furthermore, given the study design (including only women with a pre-operative diagnosis of AEH), the exact diagnostic accuracy of each ESM could not be provided. However, it should be noted that the present study included a generous sample size with a high number of hysteroscopic endometrial samplings over 18 years.

## Figures and Tables

**Table 1:** Comparison of patient characteristics between the three endometrial sampling methods.

Independent Variables	D&C (75) *n* (%)	HSC-Bio (75) *n* (%)	HSC-Res (58) *n* (%)	*p-*Value
**Age** **(median and interquartile ranges)**	54.0 (49.25–59.75)	52.0 (47.25–56.75)	56.0 (50–62)	0.094
**Menopause**	41 (54.7)	38 (50.7)	37 (63.8)	0.310
**Nulligravid**	10 (13.3)	13 (17.3)	17 (29.3)	0.060
**Smoking habit**	9 (12.0)	12 (16.0)	9 (15.5)	0.754
**Body Mass Index**	26.34 (23.03–29.29)	25.39 (22.44–29.38)	26.50 (24.16–31.0)	0.297
**Comorbidity**				0.375
Diabetes	2 (2.7)	1 (1.3)	0 (0.0)	
Hypertension	9 (12.0)	10 (13.3)	11 (19.0)	
Diabetes + Hypertension	0 (0.0)	2 (2.7)	0 (0.0)	
**Hereditary Cancer Syndromes**				0.731
Lynch S	1 (1.3)	1 (1.3)	1 (1.7)	
Colorectal cancer	1 (1.3)	0 (0.0)	0 (0.0)	
Breast and ovarian cancer S	0 (0.0)	1 (1.3)	0 (0.0)	
**Previous breast cancer**	11 (14.7)	15 (20.0)	10 (27.8)	0.688
**Tamoxifen users**				0.861
Previous users	3 (4.0)	3 (4.0)	2 (3.4)	
Current users	6 (8.0)	7 (9.3)	8 (13.8)	
**Menarche** **(median and interquartile ranges)**	12 (12.0–13.0)	12 (11.0–13.0)	12 (11.0–13.0)	0.324
**Hormonal therapy**				0.881
OC	7 (9.3)	6 (8.0)	3 (5.2)	
HRT	1 (1.3)	2 (2.7)	1 (1.7)	
**Abnormal uterine bleeding**	51 (68.0)	53 (70.7)	34 (58.6)	0.321

D&C: dilation and curettage; HSC-bio: hysteroscopically-guided biopsy; HSC-res: hysteroscopic endometrial resection; OC: oral contraceptive; HRT: hormonal replacement therapy.

**Table 2:** Comparison of endometrial cancer rate in women with atypical endometrial hyperplasia (AEH) according to endometrial sampling method.

Women with Pre-Operative AEH	Final Histology (Hysterectomy)		
	No Endometrial Cancer *n* (%)	Endometrial Cancer *n* (%)	*p-*Value
**Endometrial sampling method**			0.0037
D&C	48 (30.2)	27 (55.1)	
HSC-bio	60 (37.7)	15 (30.6)	
HSC-res	51 (32.1)	7 (14.3)	
Total	159 (100.0)	49 (100.0)	

AEH:atypical endometrial hyperplasia; D&C: dilation and curettage; HSC-bio: hysteroscopically guided biopsy; HSC-res: hysteroscopic endometrial resection.

**Table 3:** Pre- and post-test probability of endometrial cancer in women with AEH according to endometrial sampling method.

Endometrial Sampling Method	Pre-Test Probability (%)	Likelihood Ratio	95% CI	Post-Test Probability (%)
D&C	23.6	1.82	1.291 to 2.580	35.3
HSC-bio	23.6	0.81	0.509 to 1.293	19.5
HSC-res	23.6	0.44	0.216 to 0.917	11.6

AEH: atypical endometrial hyperplasia; D&C: dilation and curettage; HSC-bio: hysteroscopically guided biopsy; HSC-res: hysteroscopic endometrial resection; CI: confidence intervals.

**Table 4:** Univariate analysis comparing women with or without endometrial cancer at hysterectomy.

Women with Pre-Operative AEH	Final Histology (Hysterectomy)		
Independent Variables	No Endometrial Cancer (159) *n* (%)	Endometrial Cancer (49) *n* (%)	*p* Value
**Age (median and interquartile ranges)**	53 (48.0–57.5)	56 (50–65.0)	0.010
**Menopause**	82 (51.6)	34 (69.4)	0.028
**Nulligravid**	30 (18.9)	10 (20.4)	0.811
**Smoking habit**	25 (15.8)	5 (10.2)	0.330
**Body Mass Index**			0.025
<24.9	61 (38.4)	18 (36.7)	
25–29.9	65 (40.9)	13 (26.5)	
30–39.9	32 (20.1)	11 (22.4)	
≥40	1 (0.6)	7 (14.3)	
**Comorbidity**			0.620
Diabetes	2 (1.3)	1 (2.0)	
Hypertension	25 (15.7)	5 (10.2)	
Diabetes + Hypertension	1 (0.6)	1 (2.0)	
**Hereditary Cancer Syndromes**			0.292
Lynch S	1 (0.6)	2 (4.1)	
Colorectal cancer	1 (0.6)	0 (0.0)	
Breast and ovarian cancer S	1 (0.6)	0 (0.0)	
**Previous breast cancer**	29 (18.2)	7 (14.3)	0.523
**Tamoxifen users**			0.572
Previous users	6 (3.8)	2 (4.1)	
Current users	18 (11.3)	3 (6.1)	
**Menarche (median and interquartile ranges)**	12 (11.0–13.0)	12 (11.0–13.0)	0.850
**Hormonal therapy**			0.281
OC	14 (8.8)	2 (4.1)	
HRT	4 (2.5)	0 (0.0)	
**Abnormal uterine bleeding**	105 (66.0)	33 (67.3)	0.865

AEH: atypical endometrial hyperplasia; OC: oral contraceptive; HRT: hormonal replacement therapy.

**Table 5:** Multivariate analysis showing variables associated with cancer at final histology in women with AEH.

Independent Variables	Odds Ratio	95% CI	*p* Value
Age (criterion: >60 age of years)	1.055	1.002–1.111	0.039
BMI = 25–29.9	0.586	0.258–1.329	0.201
BMI = 30–39.9	0.775	0.302–1.988	0.597
BMI ≥ 40	19.751	2.193–177.829	0.007
Menopause = no	1.002	0.385–2.605	0.996

AEH:atypical endometrial hyperplasia; CI: confidence intervals; BMI: body mass index.

**Table 6:** Pre- and post-test probability for endometrial cancer in women with AEH and risk factors (BMI and/or age).

Endometrial Sampling Method	Pre-Test Probability (%)	Likelihood Ratio	95% CI	Post-Test Probability (%)
D&C	44.2	2.16	1.016 to 4.597	63.12
HSC-bio	44.2	1.47	0.573 to 3.778	53.81
HSC-res	44.2	0.31	0.122 to 0.814	19.96

AEH: atypical endometrial hyperplasia; D&C: dilation and curettage; HSC-bio: hysteroscopically guided biopsy; HSC-res: hysteroscopic endometrial resection; CI: confidence intervals.

## Abbreviations

AEH	atypical endometrial hyperplasia endometrial
ESM	endometrial sampling method
D&C	dilation and curettage
HSC-bio	hysteroscopically guided biopsy
HSC-res	hysteroscopic endometrial resection
EC	endometrial cancer
BMI	body mass index
